# The Musculo-Spiral Nerve in Fracture of the Humerus

**Published:** 1899-04-29

**Authors:** 


### THE MUSCULO-SPIRAL NERVE IN FRACTURE OF THE HUMERUS.

It often enough happens that the musculo-spiral nerve becomes involved in the callus formed around a fracture of the humerus, and that in this manner paralysis is set up in the muscles which the nerve supplies. But in a couple of cases reported to the Clinical Society by Mr. Clement Lucas the nerve was found to have been caught actually between the broken bones. The first case was one of a man, aged 41, who in November, 1895, had had a compound fracture of the humerus, caused by a kick from a horse. It was wired at a local infirmary. He was admitted to Guy's Hospital in May, 1896. There was then a false joint at the junction of the upper two-thirds with the lower third of the left humerus. The elbow was fixed in a semi-flexed position, the fingers and wrists were also flexed and fixed, and there was loss of sensation over the distribution of the radial nerve. Under the influence of massage, passive movements, and electricity, the movements of the elbow were restored, and some pronation, supination, and partial movement of the fingers and wrists were rendered possible, but musculo-spiral paralysis remained. On June 12th, 1896, an incision was made, and the musculo-spiral nerve was traced up to where it was found engaged between the fragments, and the wire encircling the fragments was found to include the fibrous extension of the nerve. The bone was therefore again resected and wired, and the nerve being cleared for about two inches above and below was also resected, about an inch of the fibrous part being cut away and the ends united by means of sterilised silk. Three months later it was found that the union was still incomplete, so the bone was again exposed and a screw, as well as a wire, was applied to the fractured ends, and the nerve again resected. As a result of this operation a sinus remained for some weeks. By November, however, it had closed, and the bone was firmly united, but the paralysis remained; and, although the arm became useful for carrying purposes, the nerve had not recovered when the patient was last seen. The second case was one of ununited fracture of the right humerus, with musculo-spiral paralysis, resulting from a bicycle accident five months before. At the time of the accident, and afterwards, the patient had suffered from severe pain down the back of his forearm on the outer side, extending to the thumb and forefinger. Mr. Clement Lucas made an incision between the triceps and brachialis anticus, four and a half inches in length, on the antero-external aspect of the arm, traced up the musculo-spiral nerve, and found it engaged between the fragments. The nerve was detached, resected, and united by silk, and the fragments of bone were resected and united by a screw and an encircling wire. Three weeks afterwards the patient left the hospital, and a month later it was found that he had good bony union. When last seen, however, although he had a useful limb, the power of extension at the wrist had not been recovered.

